# Exploring the Expression Differences Between Professionals and Laypeople Toward the COVID-19 Vaccine: Text Mining Approach

**DOI:** 10.2196/30715

**Published:** 2021-08-27

**Authors:** Chen Luo, Kaiyuan Ji, Yulong Tang, Zhiyuan Du

**Affiliations:** 1 School of Journalism and Communication Tsinghua University Beijing China; 2 The Faculty of International Media Communication University of China Beijing China; 3 Institute of Communication Studies Communication University of China Beijing China

**Keywords:** COVID-19, vaccine, Zhihu, structural topic modeling, medical professional, laypeople, adverse reactions, vaccination, vaccine effectiveness, vaccine development

## Abstract

**Background:**

COVID-19 is still rampant all over the world. Until now, the COVID-19 vaccine is the most promising measure to subdue contagion and achieve herd immunity. However, public vaccination intention is suboptimal. A clear division lies between medical professionals and laypeople. While most professionals eagerly promote the vaccination campaign, some laypeople exude suspicion, hesitancy, and even opposition toward COVID-19 vaccines.

**Objective:**

This study aims to employ a text mining approach to examine expression differences and thematic disparities between the professionals and laypeople within the COVID-19 vaccine context.

**Methods:**

We collected 3196 answers under 65 filtered questions concerning the COVID-19 vaccine from the China-based question and answer forum Zhihu. The questions were classified into 5 categories depending on their contents and description: adverse reactions, vaccination, vaccine effectiveness, social implications of vaccine, and vaccine development. Respondents were also manually coded into two groups: professional and laypeople. Automated text analysis was performed to calculate fundamental expression characteristics of the 2 groups, including answer length, attitude distribution, and high-frequency words. Furthermore, structural topic modeling (STM), as a cutting-edge branch in the topic modeling family, was used to extract topics under each question category, and thematic disparities were evaluated between the 2 groups.

**Results:**

Laypeople are more prevailing in the COVID-19 vaccine–related discussion. Regarding differences in expression characteristics, the professionals posted longer answers and showed a conservative stance toward vaccine effectiveness than did laypeople. Laypeople mentioned countries more frequently, while professionals were inclined to raise medical jargon. STM discloses prominent topics under each question category. Statistical analysis revealed that laypeople preferred the “safety of Chinese-made vaccine” topic and other vaccine-related issues in other countries. However, the professionals paid more attention to medical principles and professional standards underlying the COVID-19 vaccine. With respect to topics associated with the social implications of vaccines, the 2 groups showed no significant difference.

**Conclusions:**

Our findings indicate that laypeople and professionals share some common grounds but also hold divergent focuses toward the COVID-19 vaccine issue. These incongruities can be summarized as “qualitatively different” in perspective rather than “quantitatively different” in scientific knowledge. Among those questions closely associated with medical expertise, the “qualitatively different” characteristic is quite conspicuous. This study boosts the current understanding of how the public perceives the COVID-19 vaccine, in a more nuanced way. Web-based question and answer forums are a bonanza for examining perception discrepancies among various identities. STM further exhibits unique strengths over the traditional topic modeling method in statistically testing the topic preference of diverse groups. Public health practitioners should be keenly aware of the cognitive differences between professionals and laypeople, and pay special attention to the topics with significant inconsistency across groups to build consensus and promote vaccination effectively.

## Introduction

### Background

As of April 23, 2021, over 0.14 billion confirmed cases of COVID-19 and nearly 3.1 million deaths have been reported worldwide [1]. The COVID-19 vaccine has been acknowledged as one of the most effective strategies to contain the ongoing public health predicament [2]. However, what needs to be recognized is that the COVID-19 vaccine still requires cautious validation of efficacy and adverse reactions since it is a relatively innovative therapeutic intervention in development [3,4]. Owing to the intrinsic uncertainty, vaccine hesitancy and vaccine-related misinformation pervaded during the COVID-19 vaccination process [5]. Some nationwide and transnational surveys also revealed that the public’s COVID-19 vaccination intentions were suboptimal [6-8]. While numerous medical professionals have devoted themselves to vaccine development at a breakneck speed [9] and eagerly promote the massive vaccination campaign, a considerable number of laypeople expressed concerns, hesitancy, and even antagonism toward COVID-19 vaccines [5]. For instance, a recent web-based poll conducted on Twitter disclosed that more than half of the respondents doubted the safety of COVID-19 vaccines [10]. To obtain a deeper insight into the different perceptions between the professionals and laypeople toward the COVID-19 vaccine, the present study endeavors to seek the potential differentiated expressions by adopting a text mining approach on a Chinese social media platform.

### The Internet as a Pivotal Communication Space for Health-Related Issues

Web-based communication provides easy and cost-effective access to a broad audience and enables interactivity and collaborative content-sharing [11]. During the past decades, the world witnessed a drastic increase in health information on the internet, along with a pronounced tendency that both patients and caregivers are growing more likely to seek health information on the internet [12]. In the meantime, people are prone to discuss health-related issues in this virtual sphere, especially during a public health crisis [13]. For example, during the COVID-19 era, some people disclosed their disease status on the internet for help-seeking [14], and a more substantial number of people talked about their own and others' symptoms as a mere natural reaction to the threat of illness [15]. Given those features, various internet platforms serve as fertile grounds for examining the public’s perceptions of health issues or events [16]. This holds true for the vaccine issue because vaccines and vaccination are buzz topics on the internet and are encompassed by provaccine and antivaccine discourses [17,18].

Recognizing the salient characteristics of the internet, health professionals spare extensive attention to utilizing the internet to launch health campaigns, deliver health knowledge, and promote behavioral change [11]. Previous studies have summarized relevant experiences in delivering health care and health interventions with the strength of internet technologies. One representative example is that some scholars classified social media into 10 categories and put forward 4 guidelines for medical professionals to better engage in web-based health communication [19]. In reality, a series of public health institutions have implemented web-based communication strategies. The Centers for Disease Control and Prevention in the United States adopted Twitter to disseminate information, interact with the audience, and alert the public throughout the Zika epidemic [20]. In a similar vein, public health agencies in Singapore also made use of Facebook for outbreak communication and communicating the Zika epidemic strategically [21]. To cope with the COVID-19 threat, many public health agencies use social media accounts to rapidly disseminate risk messages to the public to curb contagion [22]. Except for health institutions, many medical professionals practice web-based health communication spontaneously; for instance, some doctors have joined eHealth communities to exchange medical information with patients or peers [23].

Taken together, searching and exchanging health information on the internet are common phenomena nowadays; both professionals and laypeople are critical actors in the web-based health communication environment. Since the internet has prominent advantages, including low cost, easy access, broad reach, and interactivity, it facilitates the lay public to share their health concerns, seek support, enhance their health-related knowledge, and communicate with one another. Meanwhile, professionals can develop health education and interventions on the internet. For public health researchers, diversified internet platforms can be exploited to investigate varying perceptions and expressions toward various kinds of health-related issues, especially emergent ones.

### Professionals vs Laypeople in Perceiving Health-Related Issues

An entrenched thought toward the divergence between professionals and laypeople emphasizes the knowledge chasm, which retains an inherent assumption that the laypeople lag behind professionals in their knowledge levels. A professional is always defined as someone who procured special knowledge or skills of a particular subject through deliberate training and practice, while laypeople usually lack formal training or practical experience [24]. Furthermore, an extended viewpoint believes that professionals’ judgments and perceptions are more objective and reliable than those of laypeople [24]. In health communication, we particularly underscore 2 additional significant dimensions stemming from the knowledge level disparity when discussing differences between professionals and laypeople: risk perception and attitude.

As a vaccine shrouded in uncertainty, societies worldwide are deluged with suspicions and debates about the COVID-19 vaccine’s safety [25]. All concerns are closely connected to risk perception, which denotes people’s subjective assessment of a risk’s characteristics and severity [26]. Risk perception is a compound of scientific judgment and subjective factors [27]. When it comes to differences in risk perception between professionals and laypeople, one school of thought holds that owing to the differences in knowledge reservation and established mindsets, professionals usually treat risks and uncertainties from an analytical, objective, and rational perspective. Laypeople, however, are favored to rely on hypothetical, subjective, and emotional cues when perceiving risks [28-30]. Moreover, laypeople are accustomed to amplifying risks and more susceptible to psychological factors, while professionals may underestimate the dangers and accentuate the benefits of certain controversial technologies [30,31]. Another school of thought refutes those assertations by demonstrating that professionals and laypeople are unanimously influenced by emotions, worldviews, and values when forming opinions about controversial issues [27,32]. For some medical topics, the scientific literacy advantage of professionals is not more prominent than that of laypeople [33,34].

Another dimension is attitude. According to the knowledge deficit model, the lay public’s skepticism toward innovative technologies can be attributed to their deficiency in scientific knowledge [35,36]. Besides, this model hypothesizes that the laypeople’s and professionals’ divergent opinions on the same issue can be ascribed to the public’s insufficient issue-specific knowledge [37]. Therefore, a more supportive attitude toward emerging technologies could be realized by enhancing the public’s scientific knowledge level or the so-called scientific literacy [35,38,39]. Although this model has been criticized by a series of empirical studies [40], it still influences health communication and science communication research. Recently, a study on emergency medicine influencers’ Twitter use during the COVID-19 pandemic disclosed that medicine influencers’ messages contain words with more positive and neutral emotion than those of the general public. The influencer group also has a manifest topic preference for clinical information and COVID-19 news [41].

### Using Social Media to Explore Expression Differences

As one of the most burgeoning branches of internet technologies, social media has been invested with plentiful unobtrusive and naturalistic data [42,43], which makes it suitable for examining heterogeneous discussions and perceptions toward specific health-related topics or events [16]. For instance, some pundits employed tweets to gain insights and knowledge of how people discuss the human papillomavirus vaccines [44]. Similarly, Twitter contents have also been applied to excavate public sentiments and opinions toward COVID-19 vaccines [45]. Similar studies have bolstered the notion that social media can offer valuable illumination to infoveillance, promoting vaccine uptake, and altering vaccine hesitancy. Nevertheless, it should be noted that this series of studies have often been conducted in Western contexts. As a country with an increasingly expanded proportion in social media usage, China has not gained enough scholarly attention.

Based on the aforementioned discussion, this research aims to explore expression differences between professionals and laypeople toward the COVID-19 vaccine on social media. This research topic is essential because it affords a basis for understanding perception disparities between professionals and nonprofessionals, which in turn provides insights into devising effective communication strategies between the 2 groups to promote COVID-19 vaccination compliance and coverage. Additionally, there are limited studies systematically examining expressions between laypeople and professionals [46,47]. Whether the abovementioned risk perception divergence and attitudinal difference reflect in expressions is still unknown. Our research endeavors to replenish the present lacuna by offering empirical evidence on how the 2 groups conceive medical technologies in a public health crisis.

Given China’s low visibility in the previous research scope, we focused on China. China was one of the first countries severely affected by COVID-19. After implementing a series of strict prevention and control measures, the Chinese government tamed the virus in a comparatively short period; the so-called “China’s model to combat the COVID-19” set an example for other countries to combat this global health crisis [14]. Furthermore, China has taken great strides in developing COVID-19 vaccines. For instance, the 2 Chinese pharmaceutical pioneers Sinovac and Sinopharm have undertaken tremendous vaccine production tasks and promoted their products domestically and overseas [48]. As one of the first-tier countries launching vaccines against COVID-19, the COVID-19 vaccine entered the Chinese public discussion sphere early, endowing us a unique opportunity to unravel the possible asymmetric perceptions between medical professionals and the public toward the same issue. In summary, we formulated two research questions: (1) is there any difference in expression between professionals and laypeople when discussing the COVID-19 vaccine in China? (2) What major themes about the COVID-19 vaccine emerged in the 2 groups’ expressions in the Chinese context? Do thematic disparities exist? The first question leans to the explicit layer and focuses on the primary text features. The second question leans to the implicit layer and targets the latent thematic structures. We believe that this study could develop an in-depth understanding of the differences between professionals and laypeople by synthesizing the 2 aspects.

## Methods

### Data Source

We selected a web-based question and answer (Q&A) forum to collect the research data. Zhihu [49], a Chinese equivalent of Quora, is the most popular social Q&A website in China [46]. According to Liang et al [46], Zhihu is an ideal platform to investigate differences between professionals and laypeople for 3 reasons. First, Zhihu amasses a substantial amount of user-generated content about controversial social issues. For example, as of May 12, 2021, the “COVID-19 vaccine” topic on Zhihu has garnered 762 questions. Second, Zhihu has a unique structure that facilitates interactive communication. Users can follow each other, invite others to answer questions, and reply to each other in the comments section. Third, professionals are highly visible and active on Zhihu. A significant proportion of experts could be easily distinguished by their self-reported personal details (eg, affiliation and working sector) or visual symbols bestowed by the platform (eg, a blue badge after the username) [46]. Those who specialize in particular fields and engage in sharing opinions are more likely to become influencers on Zhihu [50]. These characteristics enable us to discern professionals from laypeople cost-effectively and discover the expression incongruities between the 2 user groups on Zhihu.

To obtain as much comprehensive data as possible, one of the authors designed a Python script to crawl all questions (including extended question descriptions) and their corresponding number of answers under the “COVID-19 vaccine” topic, which is the most relevant and active topic about the COVID-19 vaccines on Zhihu. Since some questions received very few responses, we excluded those questions with less than 10 answers. Next, we adopted another self-written Python script to collect each answer’s concrete content along with each respondent’s public profile. The content serves as the core corpus of the current study, whereas the public profiles are used to determine the identity category of the respondent. Finally, 65 questions were retained for the ensuing analysis with 3196 answers under them. Multimedia Appendix 1 provides details regarding the reserved questions. Data collection was finished on March 23, 2021.

### Coding Scheme

Manual coding was applied to differentiate the 2 types of identities and classify the 65 retained questions. According to the Merriam-Webster Dictionary, a professional can be defined as someone who conforms to the technical or ethical standards of a profession [51]. Because of the inherent medical attributes of COVID-19 vaccines, we further narrowed the meaning scope of *professional* by restricting it to medical professionals. Two criteria were set to distinguish the professional identity: (1) users licensed or certified to provide health care services to natural persons (eg, physicians and pharmacists) [52] and (2) users who major or conduct research in medicine or related fields (eg, Chinese pharmacy or life sciences) [46]. Laypeople are also evaluated on the basis of two criteria: (1) users who explicitly disclose their identities, other than medical professionals and (2) users who do not divulge their identities explicitly. Identification cues are extracted from pertinent information units in the user’s public profile, including self-reported educational experience, working sectors, career history, and authentication information.

With regard to the reserved 65 questions, it is untenable to perform between-group comparisons 65 times. In other words, it is not sensible to compare professionals’ and laypeople’s expressions under each question because it would be difficult to draw a representative and systematic conclusion through repeated small-scale analysis. Therefore, we classified those questions to find out some common underlying characteristics among them. In line with previous experience [53], we carried out semiopen coding to clarify question categories. All authors discussed the classification framework back and forth on the basis of personal understanding after reviewing all questions and their descriptions. Later, we performed a pilot manual coding to confirm the rationality and applicability of the preliminary categories. The final classification comprises 5 categories (Table 1), which suit all questions well. The mapping relationships between individual questions and categories can also be found in Multimedia Appendix 1. More specifically, the 5 categories in Table 1 resonate with preceding studies. Firstly, people’s COVID-19 vaccination intention primarily hinges on the safety and side effects of the relevant vaccines [54]. COVID-19 vaccines’ efficacy and safety profile are vital for its successful deployment and the achievement of herd immunity [6,9]. Thus, “adverse reactions” and “vaccine effectiveness” are 2 indispensable categories when discussing the COVID-19 vaccine. Secondly, one study about discerning topics regarding vaccines on the internet proposed that disease outbreaks, vaccine development, vaccine studies, and vaccination guidelines emerged in web-based articles on vaccines [55]. Besides, many scholars accentuated vaccines’ nonnegligible role in preventing communicable diseases and indicate the severity and hidden threats resulting from vaccine hesitancy from a societal perspective [2,56,57]. Our remaining 3 question categories (Table 1) have significant overlap with those findings.

**Table 1:** Question categories and their meanings.

Category	Meaning
Adverse reactions	Asking about any unintended or dangerous human reactions to COVID-19 vaccines
Vaccination	Asking about COVID-19 vaccination programs, arrangements, intentions, and status quo
Vaccine effectiveness	Asking about the physiological reactions in individuals, such as the effectiveness and success signs of a specific type of COVID-19 vaccine or efficacy comparison between candidate vaccines
Social implications of the vaccine	Asking about the social consequences of the emergence and uptake of the COVID-19 vaccine, such as whether COVID-19 vaccines can achieve herd immunity
Vaccine development	Asking about details regarding the COVID-19 vaccine development process, such as performance indicators in the 3 trial phases

### Analytical Strategies

We selected traditional content analysis and automated text analysis as our research methods to address the 2 proposed research questions. Conventional content analysis aimed to distinguish the identity of each respondent through manual coding. Three authors coded 50 randomly sampled respondents in accordance with the aforementioned designated criteria in the pilot coding stage. Intercoder reliability reached an ideal state (Krippendorff *α*=.93). The 3 authors then coded the remaining respondents independently. Similarly, 3 authors coded 20 randomly selected questions to test intercoder reliability for the question category. The reliability coefficient also meets the statistical standard (Krippendorff *α*=.91).

Owing to the large volume of answers, we leveraged automated text analysis to analyze the corpus efficiently. Automated text analysis is a broad terminology for a series of natural language processing methods, including but not limited to frequency analysis, co-occurrence analysis, and topic modeling [58]. This automated approach benefits text miners in alleviating the labor-intensive task of coding texts manually. More specifically, we calculated the fundamental expression characteristics of the 2 user groups, including the answer length, distribution of attitudes, and high-frequency words [46,59]. Attitudinal analysis was completed using the up-to-date TextMind software developed by the Chinese Academy of Science, which can be regarded as the Chinese version of LIWC (Linguistic Inquiry and Word Count) [60]. TextMind is capable of inferring emotional states, intentions, and thinking styles from text through a dictionary-based approach with high reliability and validity [61].

For thematic analysis, we utilized topic modeling to probe into the thematic differences between the 2 identities. Topic modeling can investigate the hidden thematic structure of a given collection of texts [62]. As one of the cutting-edge branches in the topic modeling family, structural topic modeling (STM) allows researchers to estimate a topic model by considering document-level metadata. In other words, STM enables researchers to discover relationships between topics and metadata, such as the topic preference of distinct authors or topic fluctuation across time [63]. STM assimilates document metadata (eg, authorship and time of publication) as covariates during the generative process; it has previously been used to explore the distinct selective sharing mechanisms of different media outlets [64] and how party identification affects topic prevalence [65]. Before formal modeling, the authors conducted preprocessing to clean the corpus, including discarding punctuation, filtering out stop-words, and pruning highly frequent words. The preprocessing procedure adheres to that of a widely recognized topic modeling study [62]. STM was implemented using the stm package in R [63], while other automated text analyses were accomplished in the Python programming environment.

## Results

The first research question asks about the expression differences between professionals and laypeople. Given the 5 predefined question categories, we examine all answers under each question category and performed statistical analysis (Tables 2-5).

Compared to the answers of professionals, those of laypeople are more prevalent (Table 2). Besides, professionals are inclined to write longer answers than laypeople (Table 3). A subsequent series of 2-tailed independent-samples *t* tests confirmed this supposition by revealing that professionals’ average answer length was significantly higher in word count than that of laypeople under each question category (adverse reactions: *t*_711_=–2.335; *P*=.02; vaccination: *t*_958_=–2.401; *P*=.02; vaccine effectiveness: *t*_415_=–2.240; *P*=.03; social implications of vaccine: *t*_260_=–2.149; *P*=.04; vaccine development: *t*_842_=–4.546; *P*<.001).

**Table 2:** The number of answers posted by professionals and laypeople under 5 question categories regarding COVID-19 vaccines (N=3196).

Question category		Answers, n (%)
**Adverse reactions**		
	Professional	68 (9.54)
	Laypeople	645 (90.46)
**Vaccination**		
	Professional	104 (10.83)
	Laypeople	856 (89.17)
**Vaccine effectiveness**		
	Professional	76 (18.23)
	Laypeople	341 (81.77)
**Social implications of the vaccine**		
	Professional	25 (9.54)
	Laypeople	237 (90.46)
**Vaccine development**		
	Professional	129 (15.28)
	Laypeople	715 (84.72)

**Table 3:** Answer length of professionals and laypeople under 5 question categories regarding COVID-19 vaccines (N=3196).

Question category		Answer word count, mean (SD)
**Adverse reactions**		
	Professional	454.12 (674.09)
	Laypeople	251.83 (806.92)
**Vaccination**		
	Professional	510.67 (1191.63)
	Laypeople	225.97 (482.32)
**Vaccine effectiveness**		
	Professional	937.03 (2408.93)
	Laypeople	310.80 (619.62)
**Social implications of the vaccine**		
	Professional	765.52 (1310.93)
	Laypeople	200.10 (331.42)
**Vaccine development**		
	Professional	815.60 (1345.11)
	Laypeople	266.18 (609.15)

**Table 4:** Attitude distribution of professionals and laypeople 5 five question categories regarding COVID-19 vaccines (N=3196).

Question category			Answers with a positive attitude, n (%)		Answers with a neutral attitude, n (%)		Answers with a negative attitude, n (%)
**Adverse reactions**							
	Professional	21 (30.88)		28 (41.18)		19 (27.94)	
	Laypeople	209 (32.40)		220 (34.11)		216 (33.49)	
**Vaccination**							
	Professional	46 (44.23)		28 (26.92)		30 (28.85)	
	Laypeople	339 (39.60)		276 (32.24)		241 (28.15)	
**Vaccine effectiveness**							
	Professional	38 (50.00)		13 (17.11)		25 (32.89)	
	Laypeople	170 (49.85)		97 (28.45)		74 (21.70)	
**Social implications of the vaccine**							
	Professional	10 (40.00)		6 (24.00)		9 (36.00)	
	Laypeople	96 (40.51)		67 (28.27)		74 (31.22)	
**Vaccine development**							
	Professional	53 (41.09)		49 (37.98)		27 (20.93)	
	Laypeople	336 (46.99)		219 (30.63)		160 (22.38)	

**Table 5:** High-frequency words of professionals and laypeople under 5 question categories regarding COVID-19 vaccines.

Question category		High-frequency words^a^
**Adverse reactions**		
	Professional	RNA, Pfizer, adverse reactions, death, America, side effects, clinical trial, inject, inactivated vaccine, data
	Laypeople	America, China, Pfizer, coronavirus, RNA, death, Japan, inject, adverse reactions, country
**Vaccination**		
	Professional	coronavirus, crowd, immune, infect, clinical trial, antibody, country, adverse reactions, disease, emergency
	Laypeople	coronavirus, Russia, America, country, China, crowd, inject, clinical trial, infect, research and development
**Vaccine effectiveness**		
	Professional	RNA, coronavirus, data, protein, effective rate, infect, cell, immune, inactivated vaccine, technology
	Laypeople	RNA, China, coronavirus, data, inactivated vaccine, America, India, technology, produce, protein
**Social implications of the vaccine**		
	Professional	coronavirus, data, clinical trial, Sinovac, infect, come into the market, China, symptom, effective rate, country
	Laypeople	country, coronavirus, price, China, research and development, control, domestic, America, free of charge, crowd
**Vaccine development**		
	Professional	clinical trial, coronavirus, RNA, experiment, research, research and development, China, infect, clinic, data
	Laypeople	America, China, RNA, coronavirus, research and development, country, pregnant woman, experiment, infect, company

^a^The 10 most frequent words are listed, and words are translated from Chinese to English. Some Chinese words correspond to more than 1 English word.

Furthermore, statistical analysis revealed that a positive attitude dominated the discussion regarding COVID-19 vaccines (Table 4). A series of chi-square tests were conducted to examine the correlation between attitude and identity. The results revealed nonsignificant relationships under 4 question categories, which suggests that professionals do not differ significantly from laypeople with respect to their attitude distribution when discussing adverse reactions (*χ*^2^_2_=1.5; *P*=.47), vaccination (*χ*^2^_2_=1.3; *P*=.51), social implications of the vaccine (*χ*^2^_2_=0.3; *P*=.86), and vaccine development (*χ*^2^_2_=2.8; *P*=.25). However, for vaccine effectiveness, the correlation reached significance (*χ*^2^_2_=6.3; *P*=.04). Post hoc analysis based on the adjusted residual (AD) score revealed that laypeople were less likely to express a negative attitude (AD=–2.100), while professionals favor a negative attitude (AD=2.100) under this category.

With respect to the high-frequency words among the 2 user groups, it is evident that laypeople mentioned countries more frequently (eg, *America*, *China*, *Japan*, *Russia*, and *India*) than professionals. Professionals talked more about medical jargon (eg, *clinical trial*, *immune*, *antibody*, *cell*, and *effective rate*) than laypeople (Table 5). However, a comparison of high-frequency words barely reveals a general word use preference pattern; the latent semantic structures still require a more in-depth inspection. Thus, we performed subsequent STM to deepen our understanding of the 2 groups’ topic preferences.

The second research question makes an inquiry about the latent themes that belong to the 2 kinds of identities under the 5 categories and accompanying possible thematic differences. For an accurate and robust estimation, we took advantage of the data-driven approach to select the number of topics, which is a built-in function in the stm package [63]. Based on the semantic coherence and residual fluctuation from multiple rounds of automated tests, we determined the topic number of each question category. The detailed indicators are exhibited in Multimedia Appendix 2.

**Figure 1:**
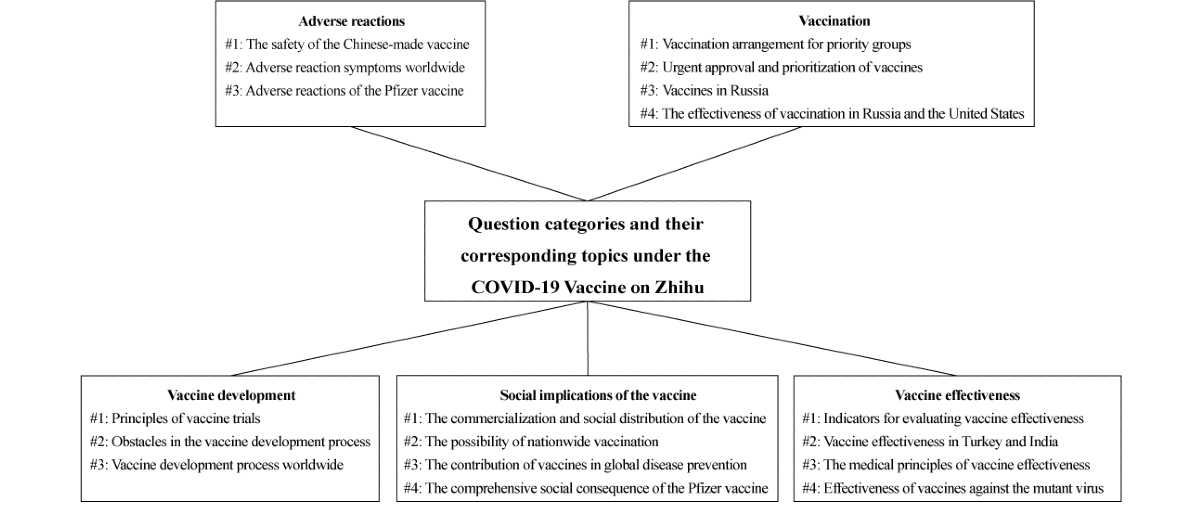
Question categories and their related topics under the COVID-19 vaccine issue on Zhihu.

According to a prior study using STM [13], the topic estimation process sticks to some assumptions. First, each document can be regarded as a mixture of latent topics, where each topic is a probability distribution of words. Second, a document is statistically generated by an iterative inference process. A topic is randomly sampled in each process, and a certain word associated with the topic is randomly drawn. The most probable topics and pertinent distributions are estimated on the basis of the given data. Although the probability distribution of words has no intuitive meaning, researchers can interpret the topic’s meaning from the relative importance (or the so-called “weight”) of words. In the current study, after executing the STM, topics were represented as collections of words. The authors labeled each topic and summarized the topic’s meaning by considering the highest-probability words and exclusive words simultaneously [63]. In STM, words with the highest probabilities and the highest frequency and exclusivity (FREX) weights are provided. A high probability implies that corresponding words are highly likely to appear under the given topic [63], while a high FREX score replenishes the high probability indicator by considering word exclusivity and frequency simultaneously [13]. Topics extracted from answers under each question category were depicted (Figure 1). Detailed topic meanings are shown in Multimedia Appendix 3. Next, we estimated the relationship between user identity and topic prevalence. The stm package illustrates those relationships with forest plots, reflecting the difference in topical prevalence between professionals and laypeople in a more expressive way.

**Figure 2:**
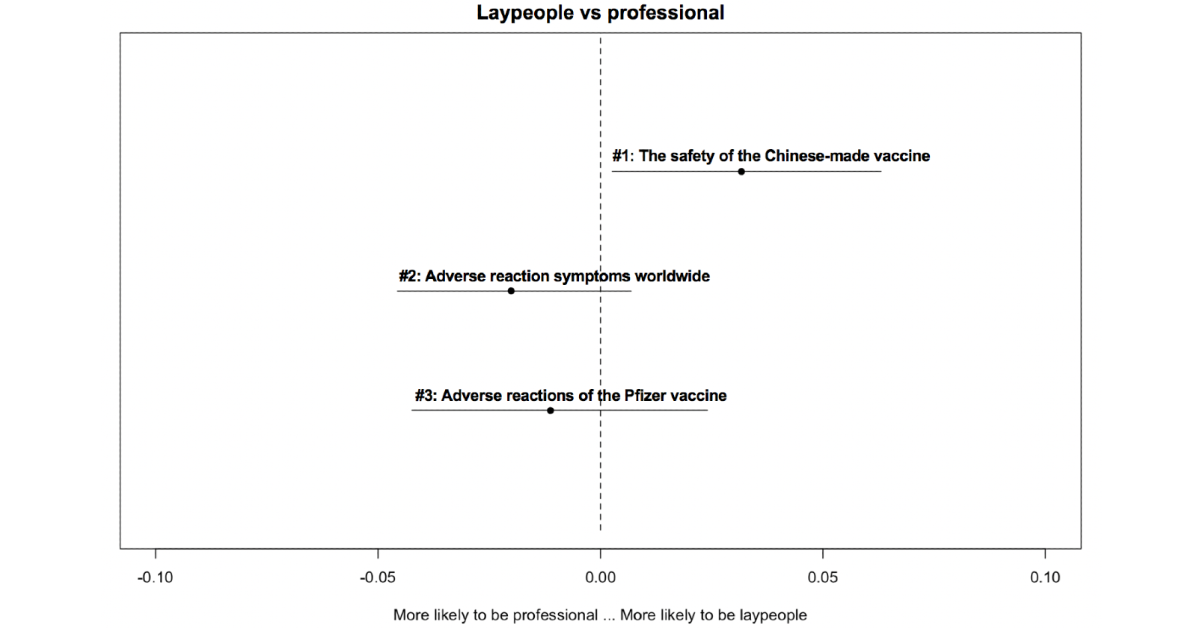
Thematic disparities between professionals and laypeople under the "adverse reactions" question category.

**Figure 3:**
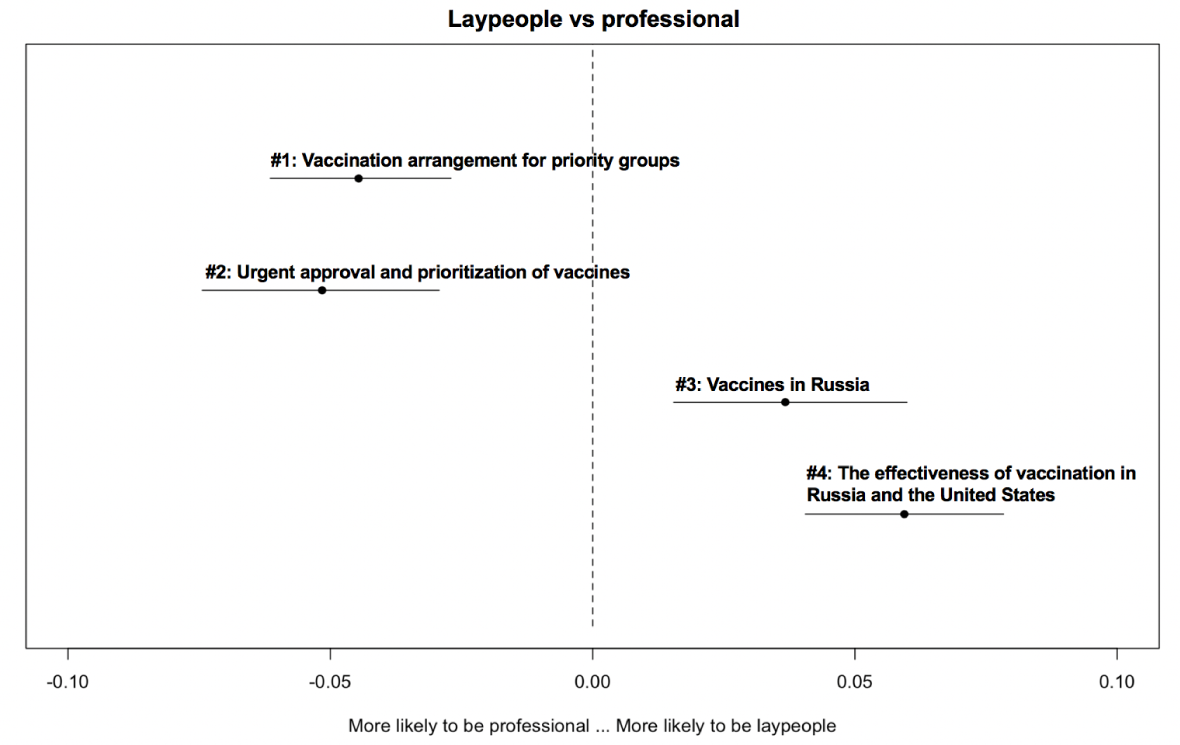
Thematic disparities between professionals and laypeople under the "vaccination" question category.

**Figure 4:**
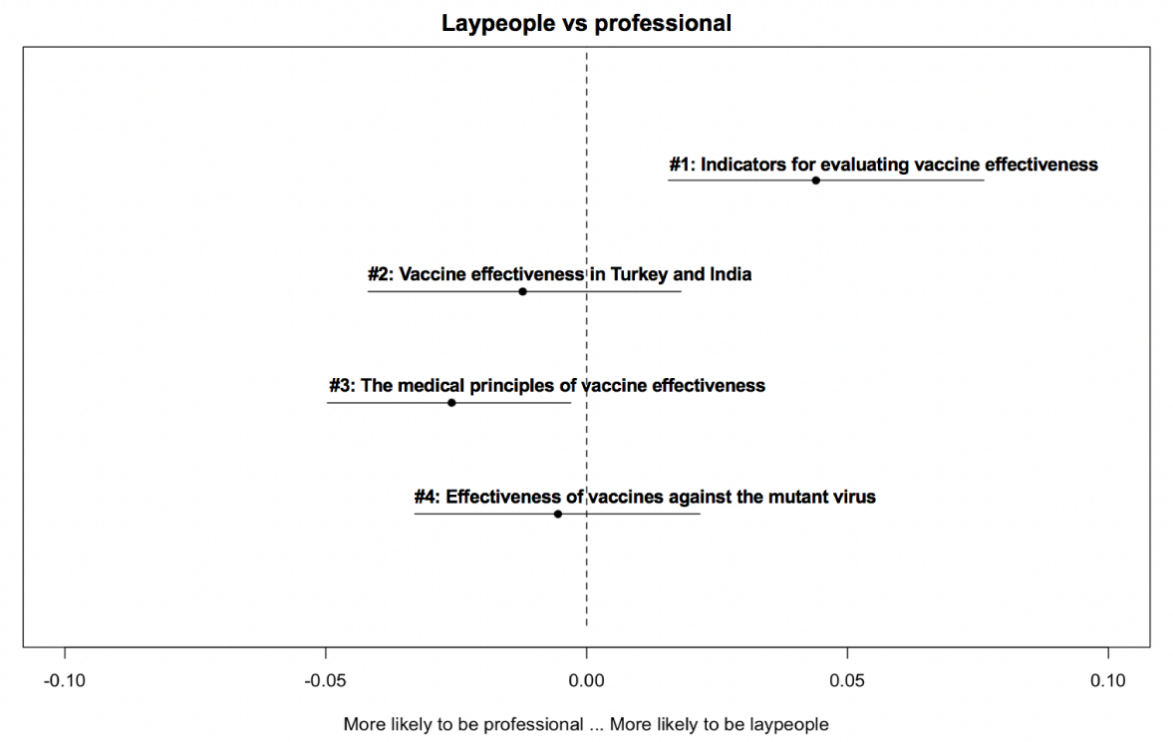
Thematic disparities between professionals and laypeople under the "vaccine effectiveness" question category.

**Figure 5:**
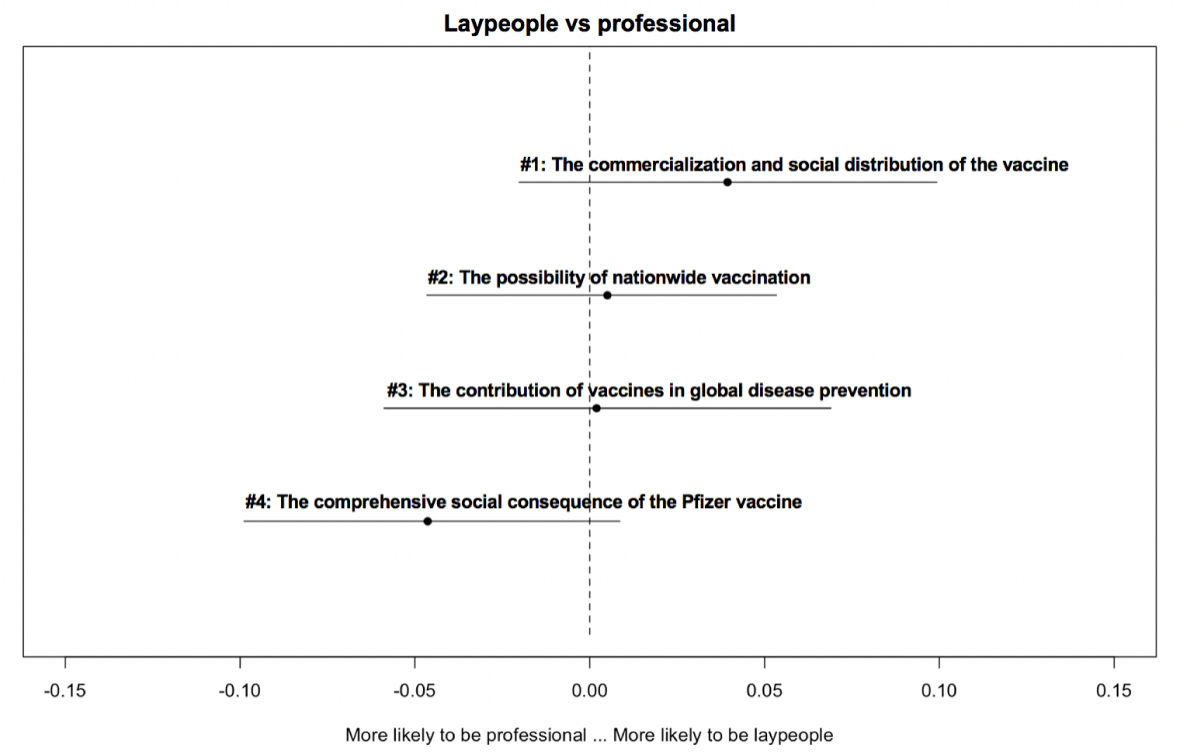
Thematic disparities between professionals and laypeople under the "social implications of the vaccine" question category.

**Figure 6:**
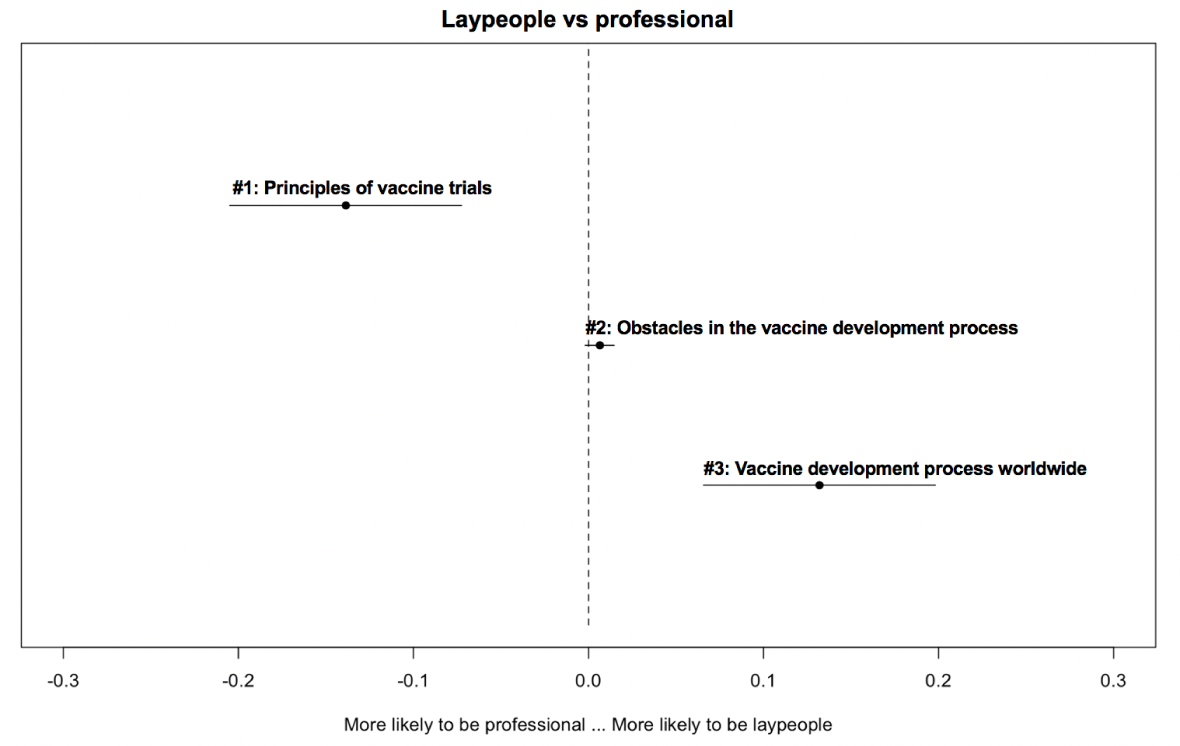
Thematic disparities between professionals and laypeople under the "vaccine development" question category.

Figures 2-6 delineate the thematic disparities between the 2 user groups under each question category. The horizontal lines represent CIs. If the CIs for each topic overlap with the dotted vertical line (indicates null effect), this implies that at the 95% CI level, professionals and laypeople do not differ from each other in adopting the topic. For the 3 topics under *adverse reactions* (Figure 2), the “safety of Chinese-made vaccine” topic is more likely to be used by laypeople (*β*=–.032; *P*=.04). For the 4 topics under vaccination (Figure 3), the two topics “vaccination arrangement for priority groups” (*β*=.044; *P*<.001) and “urgent approval and prioritization of vaccines” (*β*=.052; *P*<.001) were primarily associated with professionals. In contrast, the other 2 topics “vaccines in Russia” (*β*=–.037; *P*<.001) and “the effectiveness of vaccination in Russia and the U.S.” (*β*=–.059; *P*<.001) were more frequently adopted by laypeople. Among the 3 topics under *vaccine effectiveness* (Figure 4), 2 varied significantly across the 2 user groups. “indicators for evaluating vaccine effectiveness” topic (*β*=–.044; *P*=.003) was more likely to be mentioned by laypeople, while “medical principles of vaccine effectiveness” (*β*=.026; *P*=.03) was more inclined to be mentioned by professionals. Regarding the 4 topics under *social implications of the vaccine* (Figure 5), none of them reached significantly difference levels. Regarding the last category (Figure 6), “principles of vaccine trials” (*β*=.139; *P*<.001) was more inclined to be mentioned by professionals. Conversely, “vaccine development process worldwide” (*β*=–.132; *P*<.001) was more inclined to be mentioned by laypeople.

## Discussion

### Principal Findings

This study aimed to disentangle the expression differences between professionals and laypeople in the context of a somewhat contentious issue. To the best of our knowledge, this is one of the few studies adopting STM to analyze thematic disparities between these 2 user groups, which goes beyond previous studies that mainly relied on the hand-annotated method [46]. Moreover, there is a shortage of studies focusing on the professional-laypeople divide during the COVID-19 pandemic. Our study contributes to comprehending the expression characteristics of the 2 identities and provides us an empirical foundation for facilitating professional-laypeople communication in a web-based Q&A environment, further helps advocate authoritative voices, and corrects misinformation in a time inundated with uncertainties and risks [66].

Per our primary findings, the first arresting finding is the active participation of laypeople in the COVID-19 vaccine issue. This phenomenon, to some extent, gives credence to the previous viewpoint on the communication-facilitating effect of social media. Brossard [67] contended that the new media technologies afford the lay audience more opportunities to participate in and discuss scientific issues in a relatively straightforward way. Similarly, Peters [68] bolsters this assertion by reporting that circumstances for web-based communication substantially challenge the once quasi-monopoly status of intermediary information disseminators (eg, professional journalists and scientists) [68]. Therefore, although laypeople do not possess equivalent professional knowledge as professionals, the former are still guaranteed sufficient opportunities to discuss professional issues with professionals. In other words, the social media platforms characterize equality, openness, and plurality, which lowers the knowledge threshold and entry barrier when discussing medical issues. However, whether this frequent occurrence of laypeople equates to effective communication or fruitful dialogue between these 2 groups needs further investigation.

Aside from the extensive participation of laypeople, our study revealed additional expression differences between the 2 user groups. First, the average answer length of professionals was longer than that of laypeople. Backed with professional knowledge and practical experience, professionals are likely to elaborate their viewpoints by incorporating various evidence. This is especially true for the COVID-19 vaccine topic because COVID-19 is a typical “sudden and unexpected event” [69] with medical puzzles, and the COVID-19 vaccine still calls for rigorous clinical trials and continuous surveillance [4]. According to Zou et al [70], statistical evidence and narrative evidence are 2 major types of evidence adopted to elucidate health-related topics. Professionals are more familiar with quantitative and numerical evidence owing to their professional background and working experience. They can also invoke narrative evidence derived from daily experiences to support their views. However, laypeople lack quantitative arguments and have to depend on narratives to expound their viewpoints. Furthermore, professionals may have a more cautious and conservative mindset because of the intrinsic features in their vocational training and educational background. One representative example is professionals are not as optimistic as laypeople when talking about vaccine effectiveness on the premise that COVID-19 vaccine development is an ongoing process that requires more reliable evidence, such as the undetermined age-specific adverse effects [71].

Our results also show that professionals and laypeople analyzed the COVID-19 vaccine issue from varying perspectives. Echoing the literature review, 1 long-standing speculation in the public health field and science communication fields is that laypeople’s risk perceptions are always insufficient with regard to scientific assessments [72]. The scientific knowledge deficiency among the lay public hampers their ability to understand specific scientific issues and establish a positive attitude toward them [38,39,73]. Considering risk perception and attitude together, we prefer to believe that laypeople’s knowledge is not quantitatively lesser than or qualitatively inferior to that of professionals. Instead, the 2 user groups share some similarities but hold different thinking angles simultaneously, which is more appropriate to be marked as “qualitatively different.” First, the 2 user groups unanimously paid attention to adverse reaction symptoms worldwide, the vaccine’s effectiveness against the mutant virus, the contribution of vaccination for global disease prevention, and some other topics, which implies overlaps in their perspectives. However, considering issues related to medical expertise, such as the vaccination question category in our study, professionals accentuate arrangement and urgent approval, which are inextricably linked to public policies, and the reasonable allocation of medical resources. Laypeople prefer to care about other countries, presumably driven by the overwhelming media coverage on epidemic situations in other countries. This comparison suggests that the disparities rest in the division between professional and experiential modes of thinking, which act as 2 thinking modes toward controversial issues. The stark contrast also manifests in high-frequency word comparison and other medical-related question categories, including vaccine development and effectiveness. Second, we did not observe clear distinctions between the 2 user groups with regard to attitude under 4 question categories, which further illustrates that the attitudinal difference assumption based on knowledge level disparities is untenable in the Chinese COVID-19 vaccine context. Despite some objective gaps in knowledge acquisition between professionals and laypeople, they were both willing to treat the COVID-19 vaccines positively. Third, the “adverse reactions” category is most closely related to risk. In fact, we did not see laypeople lay excessive stress on the abnormal symptoms. This finding debunks the risk perception disparities that originated from the knowledge deficiency supposition, which implies that laypeople are not always amplifying the risks. They favor countries’ specific situations and think from living experience rather than magnifying vaccine risks or expressing suspicion regarding COVID-19 vaccines.

Regarding the social implications of the vaccines, as a category not closely linked to medical knowledge, the 2 user groups showed no significant differences. This finding indicates that the professional and experimental thinking modes lost their explanatory power when encountering the abstract issue. The social implications of COVID-19 vaccines can be broad and intricate, related to a wide range of societal dimensions. Hence, it is difficult for professionals or laypeople to lay particular emphasis on merely 1 mode. Combining the topics’ similarities and incongruities between the 2 user groups, we conclude that apart from the overlaps, the “qualitatively different” characteristic is also common on the web-based Q&A forum, which reflects different perspectives derived from knowledge background and life experience. In the context of COVID-19 vaccines, the medical-related questions are more sensitive to the influence of the “qualitatively different” feature, while more broad and abstract questions seem impervious to this feature.

### Limitations

Our analysis bears several caveats. With respect to the question categories, the COVID-19 vaccine is a multifaceted, intricate, and context-dependent issue associated with copious aspects [5]. Some question categories, such as vaccines and international relations, are omitted in this study and hence need to be further explored in future studies. Besides, the inclusion of longitudinal perspectives in this text mining study would yield more intriguing findings. For instance, with the development of the COVID-19 pandemic, will the thematic differences between these 2 user groups become wider or narrower? A dynamic and longitudinal approach would undoubtedly advance our comprehension of the ongoing COVID-19 vaccine issue and help curb this public health emergency. Furthermore, 1 aspect that cannot be dismissed is that the answers, of both professionals and laypeople, were largely hinged on the characteristics of the questions. Thus, the topic distribution may be confined within the questions’ scopes. Future studies could focus on other social media platforms (eg, Twitter and Sina Weibo) to obtain a more holistic discursive landscape, which may be more topic-rich owing to the absence of designated questions.

### Conclusions

This study provides an overview of opinion patterns and scrutinizes the expression differences between professionals and laypeople toward the COVID-19 vaccine. In terms of quantity, laypeople are the dominant discussants in the web-based Q&A forum Zhihu. Regarding expression differences, the professionals preferred writing longer answers than laypeople; they also showed a conservative stance in vaccine effectiveness and tended to mention medical terminologies in their discussions. By exerting the power of STM, as a valuable tool under unsupervised machine learning, we outlined the topics under each question category, along with the topic preference of the 2 groups. In a nutshell, professionals paid more attention to the medical principles and professional standards nested in discourses on COVID-19 vaccines. In contrast, laypeople showed solicitude explicitly for vaccine-related issues at the national and global levels, and to the safety of the Chinese-made vaccine. The 2 user groups shared some common grounds and manifested distinct concerns within the COVID-19 vaccine context.

We believe that this study has some implications and merits. First, public health scholars should be keenly aware of expressions and discussions on web-based Q&A forums, which were comparatively overlooked in prior infoveillance or infodemiology studies [74]. Q&A forums such as Zhihu or Quora make a clear distinction between professionals and laypeople, thus providing researchers with opportunities to explore the professional-laypeople incongruities in discursive styles and core topics. These dimensions may further facilitate addressing the underlying “distance” or “gap” between the 2 user groups [68]. Second, extant studies germane to COVID-19–related topic modeling widely to probe into public concerns and public awareness [75,76]. However, there is a paucity of studies on the thematic differences among various identities. Our attempts using STM provide a viable solution to discover the nuanced differences between distinct identities, unfolding some particular advantages over traditional topic modeling. Third, for public health educators, effective professional-laypeople communication does not need to focus on all underlying topics. Considering the “qualitatively different” characteristic, practitioners should focus on discussing topics that are significantly inconsistent across different identities and strive to mitigate misunderstanding while generating consensus on those topics. For example, some scholars found that popular conspiracies on Chinese social media, which are related to the pandemic’s origin, are about whether country actors intentionally developed SARS-CoV-2 in the laboratory or as bioweapons [77]. Since laypeople are highly concerned with COVID-19 vaccines in foreign countries, public health practitioners must closely scrutinize relevant discussions to guard against the emergence of vaccine-related rumors, conspiracies, or hate speech and strive to create an atmosphere for a rational discussion.

## Appendix

Multimedia Appendix 1 Question categories and corresponding questions with their number of answers.

Multimedia Appendix 2 Using semantic coherence and residual fluctuation to determine the number of topics (k) under each question category.

Multimedia Appendix 3 Topics, topic meaning, and corresponding keywords under each question category.

## Abbreviations

AD: adjusted residual
FREX: frequency and exclusivity
Q&A: question and answer
STM: structural topic modeling
