# Supplementary material for: Exploring the Expression Differences Between Professionals and Laypeople Toward the COVID-19 Vaccine: Text Mining Approach
Source: J Med Internet Res. 2021 Aug 27;23(8):e30715. doi: 10.2196/30715 (PMC8404777; doi:10.2196/30715)
Supplement: Multimedia Appendix 1 [file jmir_v23i8e30715_app1.doc]

**Multimedia Appendix 1**

Question categories and corresponding questions with their number of answers.

| ID | Category | Question | Number of answers |
| --- | --- | --- | --- |
| 1 | Adverse reactions | How do you view the case that an American nurse fainted after taking the Pfizer vaccine? Is this an adverse reaction to this COVID-19 vaccine? Would this be an important issue? | 318 |
| 2 | Adverse reactions | How do you view the death of 23 older people in Norway after taking the Pfizer vaccine? How do you view Japanese experts' suggestion that Chinese-made vaccines should be considered? | 163 |
| 3 | Adverse reactions | What do you think about the facial paralysis symptom of four volunteers after taking the Pfizer vaccine? Will it affect the follow-up vaccination process? | 46 |
| 4 | Adverse reactions | How do you evaluate that an American nurse said the Pfizer vaccine has severe adverse reactions? | 41 |
| 5 | Adverse reactions | Is it true that WHO does not recommend taking Moderna's COVID-19? What's the actual situation? | 20 |
| 6 | Adverse reactions | What do you think of American CDC's record that 1,170 recipients dead after taking the COVID-19 vaccine? | 63 |
| 7 | Adverse reactions | What do you think about Japan's promise of compensating 2.7 million dollars for the death after taking the COVID-19 vaccine? How to determine the direct relationship between COVID-19 vaccination and death? | 23 |
| 8 | Adverse reactions | How do you think of the rumor spread by the Christian Daily that a quarter of recipients in a German nursing home dead after taking the Pfizer COVID-19 vaccine? | 17 |
| 9 | Adverse reactions | Many European countries have suspended adopting the COVID-19 vaccine developed by AstraZeneca due to suspected blood clots in recipients. What's the current situation? What are the possible causes for this? | 22 |
| 10 | Social implications of the vaccine | The domestically-produced inactivated COVID-19 vaccine is expected to enter the market at the end of December, and the total price of two doses is less than 1,000 yuan. Will the epidemic be effectively controlled? | 184 |
| 11 | Social implications of the vaccine | There are more than 50 million confirmed COVID-19 cases worldwide. With the advent of Thanksgiving and Christmas, will the epidemic be worse? When will the vaccine become widely available? | 22 |
| 12 | Social implications of the vaccine | Sinovac's inactivated vaccine has been approved for entering the market on February 6th, 2021. What are the positive implications? | 23 |
| 13 | Social implications of the vaccine | Wuhan has launched the urgent vaccination of the COVID-19 vaccine. How will this arrangement influence the prevention and control of the pandemic? | 12 |
| 14 | Social implications of the vaccine | Pfizer claimed that it had developed the COVID-19 vaccine successfully with a prevention rate of 90%. What global implications will this cause? | 11 |
| 15 | Social implications of the vaccine | How long will it take to end the epidemic after the COVID-19 vaccine comes out? | 10 |
| 16 | Vaccination | How do you think Putin said that one of his daughters had taken the first COVID-19 vaccine registered in Russia on August 11th? | 324 |
| 17 | Vaccination | Twenty-three older people in Norway died after taking vaccines made by Pfizer and BioNTech. Is mRNA vaccine suitable for older people? | 90 |
| 18 | Vaccination | China has officially launched the emergency use of the COVID-19 vaccine on July 22nd. What scopes will it cover? | 63 |
| 19 | Vaccination | The COVID-19 vaccine is coming. Will you get vaccinated? | 64 |
| 20 | Vaccination | The National Health Commission said that the critical population would be vaccinated against the COVID-19. Who constitutes the critical population? What are the precautions for vaccination? | 44 |
| 21 | Vaccination | WHO claimed that we could end the COVID-19 pandemic only if 70% of the world's population get vaccinated. Why is the threshold value 70%? What are the current difficulties in vaccination? | 33 |
| 22 | Vaccination | When are you going to take the COVID-19 vaccine? | 33 |
| 23 | Vaccination | Seven million doses of inactivated vaccine have been allocated to all provinces in China, and the vaccine's safety has been proved. Does anyone around you already been vaccinated? | 25 |
| 24 | Vaccination | How do you think Biden, the president-elect of the United States, vaccinated against COVID-19 in front of the public? | 30 |
| 25 | Vaccination | How do you think about Sinopharm opened COVID-19 vaccination appointments in Beijing and Wuhan? | 29 |
| 26 | Vaccination | George Fu Gao said that the COVID-19 vaccine is expected to employ for emergency use in September. How do you understand his words? | 23 |
| 27 | Vaccination | Professionals claimed that they do not recommend taking the COVID-19 vaccine and HPV vaccine at the same time. What are the possible risks? Is there anything that needs to be aware of? | 21 |
| 28 | Vaccination | What is the difference between vaccinating two doses of the COVID-19 vaccine within one day and 14 days apart? | 13 |
| 29 | Vaccination | What do you think of Reuters' news that Trump announced the COVID-19 vaccine would hand over next week? | 14 |
| 30 | Vaccination | My workplace is counting the employees who registered for vaccination voluntarily. However, the vaccination costs 500 yuan. Is it necessary for me to register? | 13 |
| 31 | Vaccination | Are you willing to take the COVID-19 vaccine if it is available? | 11 |
| 32 | Vaccination | There is a large number of people infected with hepatitis B in China. Could people with hepatitis B take the COVID-19 vaccine? | 10 |
| 33 | Vaccination | Wuhan has started the COVID-19 emergency vaccination targets at the critical population. Who constitutes the critical population? How could the non-critical population get vaccinated? | 10 |
| 34 | Vaccination | How do you view Wenhong Zhang's prediction that the COVID-19 vaccination will be started at the end of this year or the beginning of the following year? | 10 |
| 35 | Vaccination | America ranks top one worldwide in COVID-19 vaccination after 90 million Americans got vaccinated. How do you think about this? | 81 |
| 36 | Vaccination | Why foreign countries prior older people for COVID-19 vaccination while China excludes older people first? | 19 |
| 37 | Vaccine development | WHO said that the COVID-19 vaccine is expected to be ready within 18 months. Why does vaccine development always take a long time? Why is it still necessary? | 132 |
| 38 | Vaccine development | Johnson & Johnson suspended the COVID-19 vaccine research due to unexplained symptoms that happened to one participant. What might have happened? What is the prospect of the follow-up research? | 118 |
| 39 | Vaccine development | China's inactivated COVID-19 vaccine has been approved for entering the UAE market, and the research data showed that the effective rate is 86%. What impacts will it cause? | 76 |
| 40 | Vaccine development | China National Biotech Corporation has officially disclosed their phase III clinical trial data of the inactivated COVID-19 vaccine and applied for market access. What influences will this have on fighting against the current epidemic? | 69 |
| 41 | Vaccine development | What's your opinion about Trump's declaration that the United States has begun to develop the COVID-19 vaccine at the press conference held on January 11th? | 39 |
| 42 | Vaccine development | How do you think about WHO's scientists claimed that China's COVID-19 vaccine had been proven effective? Does this mean we are not far from vaccination? | 32 |
| 43 | Vaccine development | What do you think of the failure of Oxford's animal test on the COVID-19 vaccine? Oxford once claimed that its COVID-19 vaccine could be put in use in September ideally. | 28 |
| 44 | Vaccine development | How do you think that the America-based pharmaceutical corporation Moderna has successfully developed the mRNA vaccine and carried out the clinical trials? | 22 |
| 45 | Vaccine development | The Lancet published the first human trial result of Wei Chen's team, which announced that their COVID-19 vaccine is safe and able to induce the immune response. How do you think of that? | 15 |
| 46 | Vaccine development | How do you think of the result that the Pfizer vaccine's protective efficacy may be far lower than expected, according to the initial data of vaccination in Israel? | 17 |
| 47 | Vaccine development | The first animal trial result of the COVID-19 vaccine has been published, confirming the safety and effectiveness of the vaccine. What are the implications of this for vaccine development? | 14 |
| 48 | Vaccine development | Moderna announced on May 18th that its COVID-19 vaccine performed better than expected in early human trials? What do you think of it? | 11 |
| 49 | Vaccine development | Will the individual or team win the Nobel Prize in Medicine because of developing the COVID-19 vaccine? | 13 |
| 50 | Vaccine development | How to evaluate Lancet's claim that the Chinese vaccine can induce the immune response rapidly? | 10 |
| 51 | Vaccine development | Pfizer plans to recruit nearly 4,000 pregnant women from the United States, Argentina, and Brazil for the COVID-19 vaccine trial. How do you think about this? | 200 |
| 52 | Vaccine development | How to evaluate the U.K.'s approval of "the COVID-19 human challenge trial" for vaccine testing? What are the implications or consequences of this? | 48 |
| 53 | Vaccine effectiveness | Which one is better? The inactivated COVID-19 vaccine or the mRNA COVID-19 vaccine? | 134 |
| 54 | Vaccine effectiveness | Turkey announced that the Sinovac vaccine from China is safe and effective, with an effective rate reaching 91.25%. What information should we pay attention to? | 56 |
| 55 | Vaccine effectiveness | How do you think that Moderna's COVID-19 vaccine's effective rate reaches 94.5%? | 39 |
| 56 | Vaccine effectiveness | How do you view the possible immune escape phenomenon of coronavirus 501.v2 found in South Africa? Does this mean the current COVID-19 vaccines or immunotherapies are no longer effective? | 34 |
| 57 | Vaccine effectiveness | The effective rate of Novavax's COVID-19 vaccine reached 89% in the United Kingdom during the experimental stage. What are the differences between Novavax's vaccine and other vaccines? | 25 |
| 58 | Vaccine effectiveness | Why don't we explore the Chinese pharmacy instead of developing the COVID-19 vaccine? | 28 |
| 59 | Vaccine effectiveness | Why the effective rate of Novavax's vaccine reaches 89.3% in the United Kingdom while the rate only reaches 49.4% in South Africa? | 16 |
| 60 | Vaccine effectiveness | How do you think the U.K. media reported that the effective rate of Pfizer vaccine reached 99.96% in Israel based on a 0.7 million recipient scale? | 20 |
| 61 | Vaccine effectiveness | Many countries are competing to buy COVID-19 vaccines developed in India. Does India's COVID-19 vaccine quality really good? | 18 |
| 62 | Vaccine effectiveness | What do you think of Oxford's COVID-19 vaccine effective rate reaches 70.4%? And the effective rate could approach 90% after shooting two doses? How does Oxford's vaccine compare with Pfizer and Moderna? | 15 |
| 63 | Vaccine effectiveness | The COVID-19 vaccine has come out. Do people fully protected against COVID-19 after vaccinated? | 11 |
| 64 | Vaccine effectiveness | How do you think that the Indian official got infected after taking the local-made vaccine? What's the actual effect of India's COVID-19 vaccine? | 11 |
| 65 | Vaccine effectiveness | Why do so many countries encourage taking the America-made COVID-19 vaccine, given the extremely intensive epidemic situation in the United States? | 10 |
