# Supplementary material for: Exploring the Expression Differences Between Professionals and Laypeople Toward the COVID-19 Vaccine: Text Mining Approach
Source: J Med Internet Res. 2021 Aug 27;23(8):e30715. doi: 10.2196/30715 (PMC8404777; doi:10.2196/30715)

**Multimedia Appendix 2**

Using semantic coherence and residual fluctuation to determine the number of topics (k) under each question category.

1. Category 1: Adverse reactions (select k = 3)


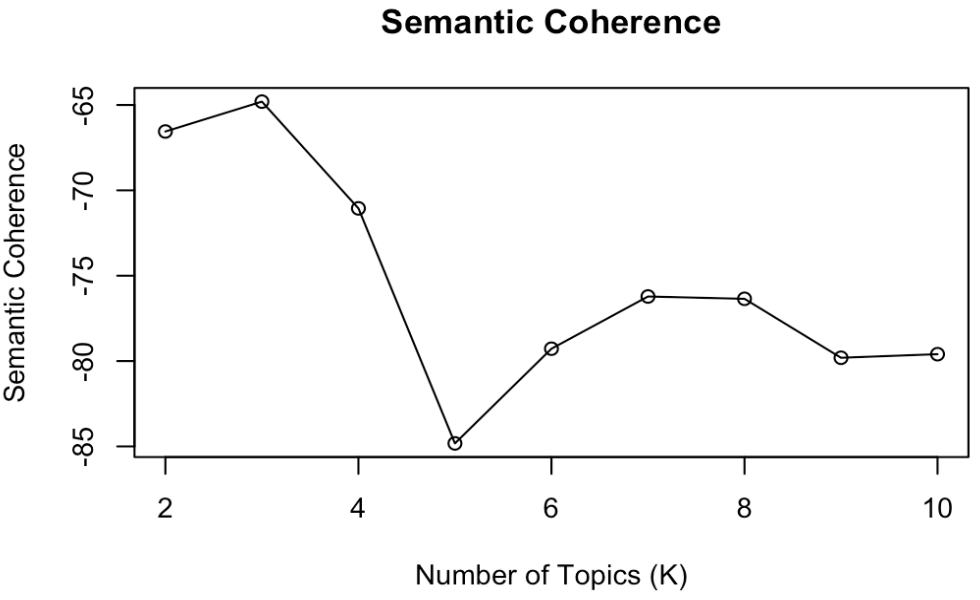

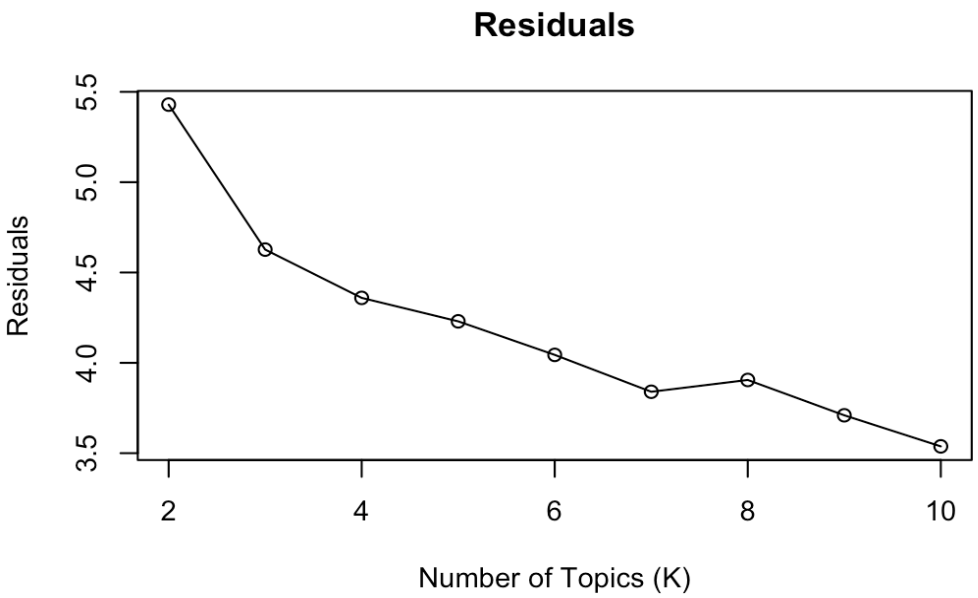


2. Category 2: Vaccination (select k = 4)


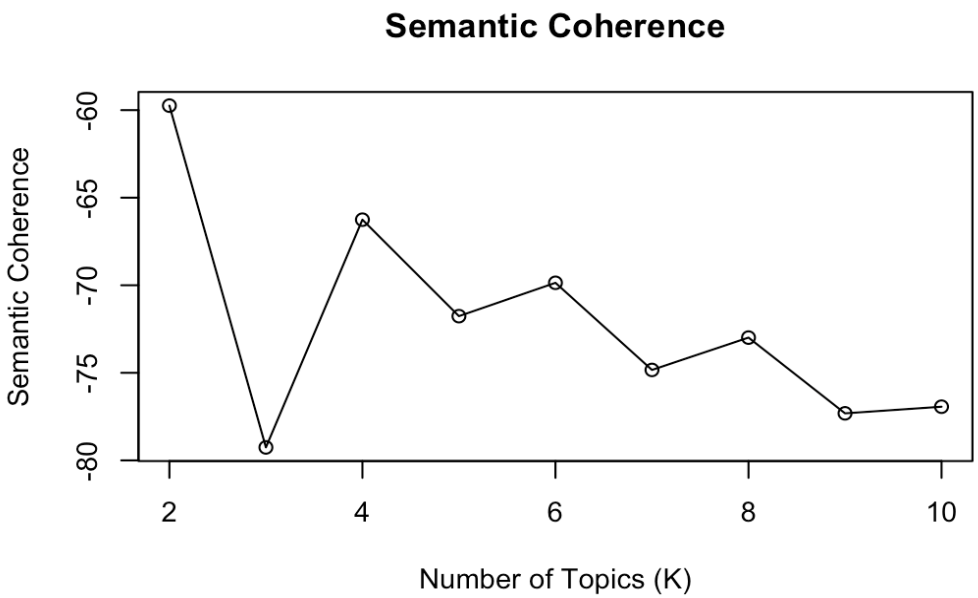


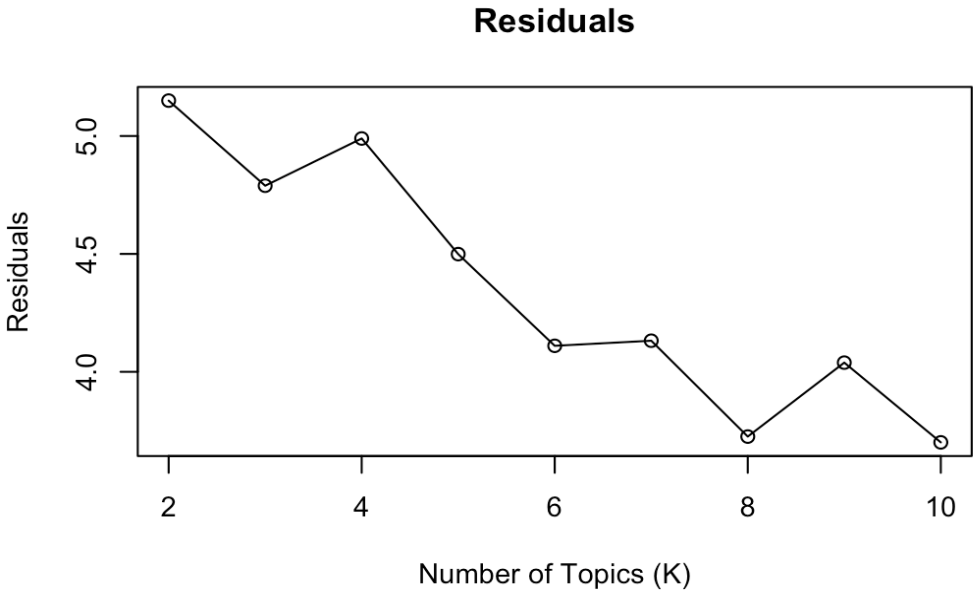


3. Category 3: Vaccine effectiveness (select k = 4)


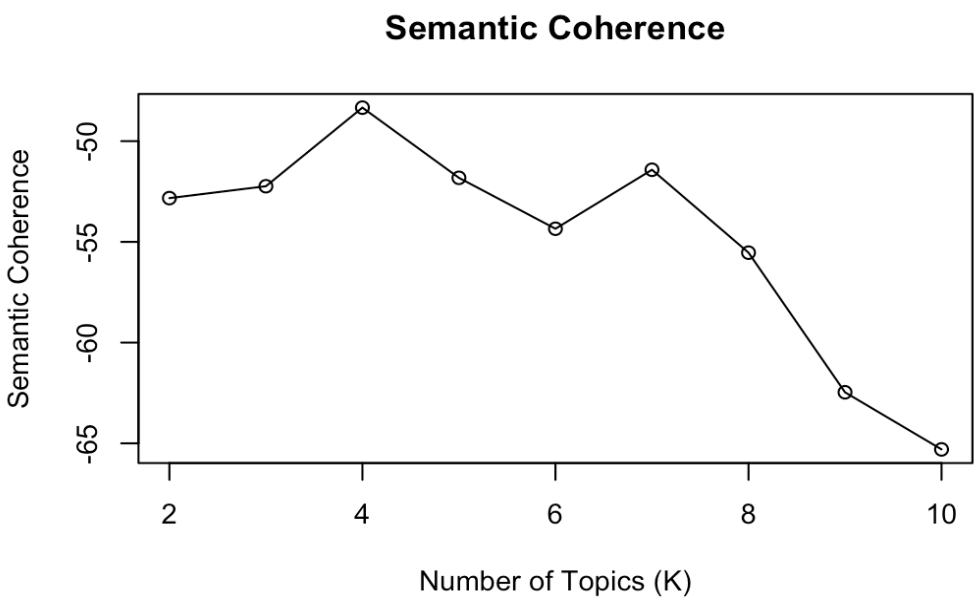


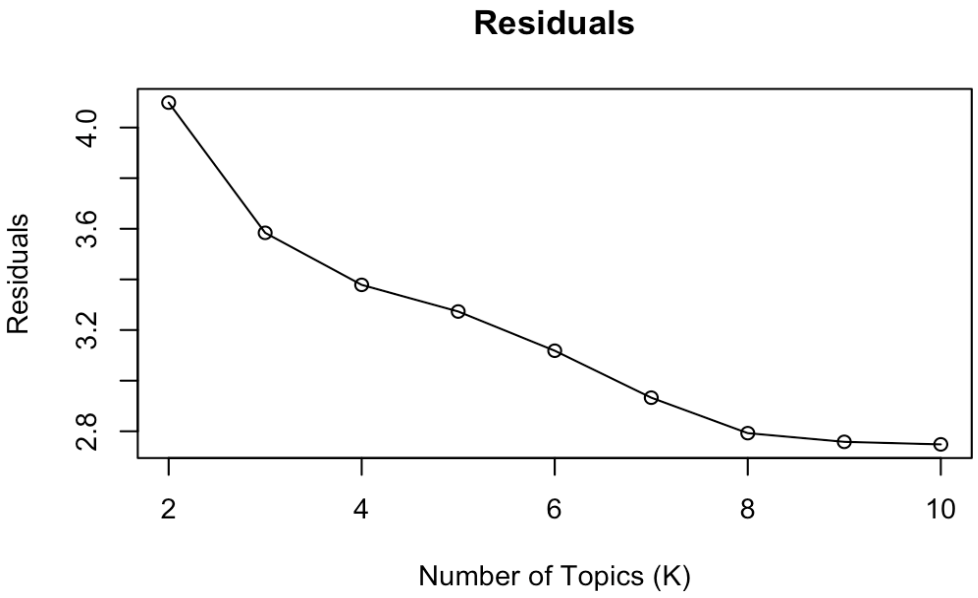


4. Category 4: Social implications of the vaccine (select k = 4)


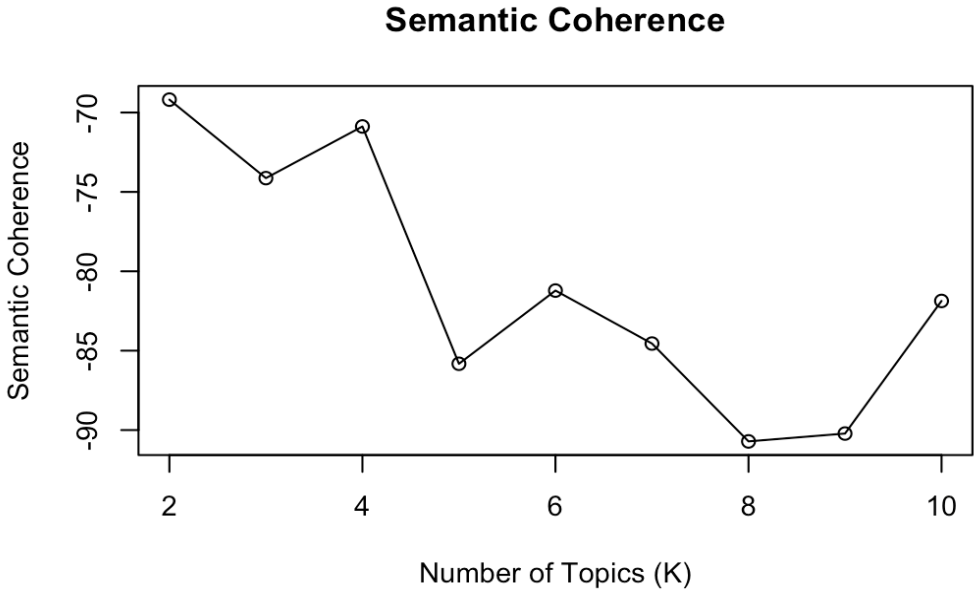


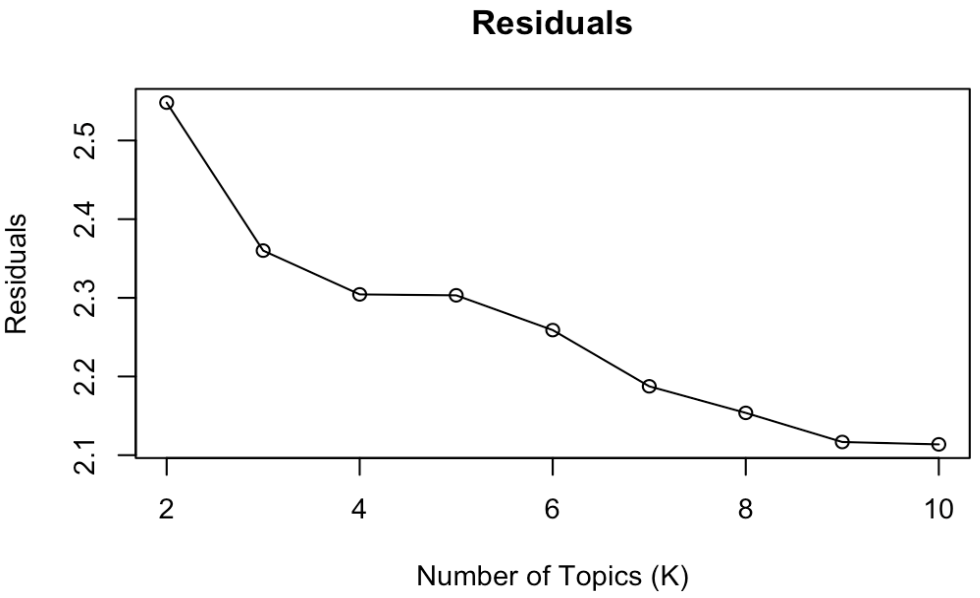


5. Category 5: Vaccine development (select k = 3)


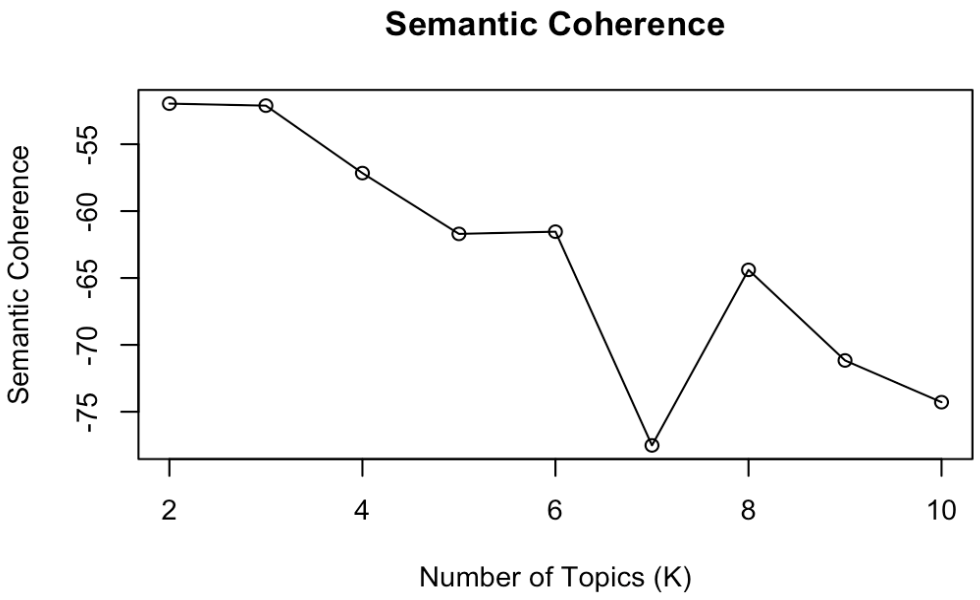


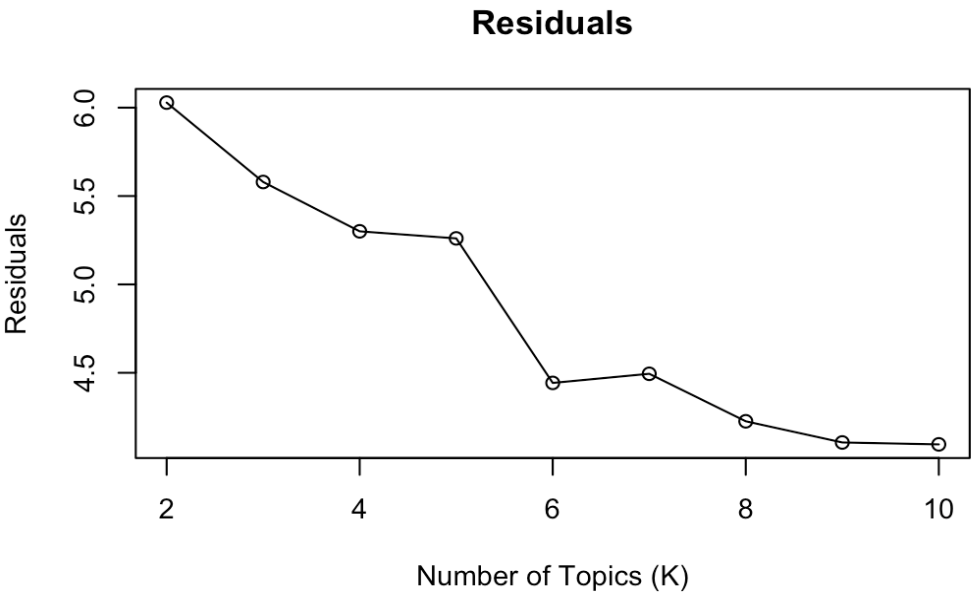

Supplement: Multimedia Appendix 2 [file jmir_v23i8e30715_app2.doc]
