# Supplementary material for: Exploring the Expression Differences Between Professionals and Laypeople Toward the COVID-19 Vaccine: Text Mining Approach
Source: J Med Internet Res. 2021 Aug 27;23(8):e30715. doi: 10.2196/30715 (PMC8404777; doi:10.2196/30715)
Supplement: Multimedia Appendix 3 [file jmir_v23i8e30715_app3.doc]

**Multimedia Appendix 3**

Topics, topic meaning, and corresponding keywords under each question category.

| Category | Corresponding topics | Topic meaning | Keywords (Top 10)^a^ |
| --- | --- | --- | --- |
| Adverse reactions | #1: The safety of the Chinese-made vaccine | The safety of vaccines made in China | China, death, inject, harmful, probability, safety, trial, expert, case, patient |
|  | #2: Adverse reaction symptoms worldwide | Specific symptoms of adverse reactions after inoculation | adverse reaction, side effect, Japan, fainting, nurse, facial paralysis, Norway, Israel, thrombus, AstraZeneca |
|  | #3: Adverse reactions of the Pfizer vaccine | Adverse reaction reports after taking the Pfizer vaccine | America, Pfizer, inactivated vaccine, risk, the old, allergy, infect, death rate, symptom, the United Kingdom |
| Vaccination | #1: Vaccination arrangement for priority groups | Arrangements for vaccinating the priority groups which are susceptible to COVID-19 | country, infect, immune, the old, staff, world, doctor, immunity, interval, overseas student |
|  | #2: Urgent approval and prioritization of vaccines | The urgent approval and emergency use of certain COVID-19 vaccines to ease the contagion | China, inactivated vaccine, global, urgency, prioritize, approve, high-risk, high-stake, staff, exposure |
|  | #3: Vaccines in Russia | Vaccines and vaccination in Russia | Russia, clinical trial, safety, research, Putin, crowd, daughter, efficacy, epidemic prevention, president |
|  | #4: The effectiveness of vaccination in Russia and the United States | Performances of COVID-19 vaccines in Russia and America | America, Putin, trial, effect, control, human body, develop, mutation, the public, Trump |
| Vaccine effectiveness | #1: Indicators for evaluating vaccine effectiveness | Concrete measurements of the efficacy of candidate vaccines | research, RNA, safety, time, data, dosage, messenger, protection rate, pathogen, cell |
|  | #2: Vaccine effectiveness in Turkey and India | Performances of candidate vaccines in Turkey and India | India, Turkey, corporation, effective rate, clinical trial, inject, effect, condition, symptom, event |
|  | #3: The medical principles of vaccine effectiveness | The medical criteria to evaluate the effectiveness of vaccines | virus, data, antibody, cell, case, placebo, strain, report, immune escape, control group |
|  | #4: Effectiveness of vaccines against the mutant virus | The efficacy of current vaccines for mutated viruses | variation, mutation, the United Kingdom, effectiveness, lower, side effect, therapeutic effect, change, prevention, neutralize |
| Social implications of the vaccine | #1: The commercialization and social distribution of the vaccine | The process of commercialization, circulation, and allocation of vaccines | price, free of charge, cost, protection rate, group, transportation, government, cold chain, output, distribution |
|  | #2: The possibility of nationwide vaccination | The probability of promoting vaccines to the whole public | immune, effectiveness, the public, human being, region, city, economic, infected, cheap, the whole people |
|  | #3: The contribution of vaccines in global disease prevention | How the vaccines help to the prevention of the COVID-19 pandemic | China, global, inactivated vaccine, Wuhan, prevention, effective rate, Russia, epidemic prevention, end, Brazil |
|  | #4: The comprehensive social consequence of the Pfizer vaccine | A plethora of social implications of the Pfizer vaccine | America, Pfizer, protection, approve, safety, technology, population, price, overseas student, probability |
| Vaccine development | #1: Principles of vaccine trials | The technical details behind vaccine trials | pregnant woman, trial, clinical trial, antibody, experiment, cell, protein, immune system, immunoreaction, induce |
|  | #2: Obstacles in the vaccine development process | Various constraints when developing COVID-19 vaccines | environment, Brazil, face, war, pressure, the poor, poor country, abortion, distrust, doubt |
|  | #3: Vaccine development process worldwide | Progress of vaccine development on a global scale | China, America, Sinopharm, corporation, Pfizer, inactivated vaccine, global, come into the market, United Arab Emirates, Moderna |

^a^Words are translated from Chinese to English; some Chinese words correspond to more than 1 English word.
